# Topics of the Month

**Published:** 1893-03

**Authors:** 


### TOPICS OF THE MONTH.

It is strange that a modern city should be compelled to tolerate a nuisance that is coming to be well nigh intolerable. In this latter part of the nineteenth century, which is fraught with a high order of civilization in everything, excepting certain bigotries which still cling around the church, it is a pity that the latter cannot give up some of its idols. We have no quarrel with sects or beliefs, for we prefer that every man and woman shall have the freest latitude in this regard, but we do think that it is high time that any church that proposes to ring its bells to the annoyance and discomfort of the patient people, should be brought to the bar of justice for its infraction of the commonest rules of propriety. Let anyone lie ill for a few weeks within the range of St. Michael's Church, and hear the intolerable ding of its bells at an early hour, when invalids need rest and sleep, and we think they will acknowledge the justice of every word we have said, and even that we have been too lenient in our charge.

We call upon the Health Commissioner to invoke the aid of the courts to suppress this nuisance, which has become a stench in the nostrils of decent people. It may be true that politicians are afraid to handle it, but we think the Health Commissioner has mettle enough in him to suppress anything which will come within the range of a sanitary nuisance.

Of what earthly good can it be to ring the church bells at five or six o'clock in the morning, and so on during all hours of the day, when everybody nowadays carries a watch, and every house is full of timepieces that indicate when services are held? We speak earnestly because we are the victims of this very nuisance. One would suppose that the good Bishop Ryan, if appealed to, would put an end to it, but we understand that he declines to do so. Therefore, we invoke the health authorities on the subject.

---

The annual banquet given by the Department of Health, at the Tifft House, on Friday evening, February 17, 1893, was a most successful gathering of convivial friends. The menu was everything that could be desired, the speeches were excellent, and the poetry was unique. These annual social meetings serve to bind together a class of officials who need recreation, and who work all the better for the health of the community because they work unitedly and agree socially.

The Columbia Daily Calendar has been received, and, as usual, fills an important place on the desk of every one fortunate enough to possess it. The day of the week, of the month, and of the year is given, and on each leaf is a short plea for outdoor exercise on a bicycle. The mechanism of this calendar is such as to make it one of the most useful that we have seen. It is issued by the Pope Manufacturing Co., of Boston, New York, and Chicago.

---

Clinical Report on Insanity is the title of a brochure published by the medical staff of the Maryland Hospital for the Insane. This is a reprint from the ninety-fifth annual report of the hospital, and is one of the most interesting publications that has been issued by such an institution within our knowledge. Its contents are as follows: (1) The Relation of Pelvic Disease to Psychical Disturbances in Women, by Geo. H. Rohé, M. D., Superintendent; (2) A Case of Trephining for Insanity, by J. Percy Wade, M. D., Assistant; (3) A Case Showing the Relation of Kidney Disease to Insanity, by Milton D. Norris, M. D., Clinical Assistant; (4) Acute Delirious Mania Probably Depending upon Septic Absorption, by Fred Caruthers, M. D., Clinical Assistant; (5) Results Obtained with Sulfonal and Hyoscine in the Treatment of the Insane, with Report of Cases, by John H. Scally, M. D., Clinical Assistant. Dr. Rohé's paper has already been published in the *American Journal of Obstetrics*, and has, no doubt, been widely read. We have already referred to this subject in a previous issue when noticing the report of the Eastern Michigan Asylum. The other papers also are of great interest, and we commend their careful reading to all interested on the subject of insanity or psychical disturbances of any form.

---

The Directors of the Carnegie Laboratory announce that they have arranged for short courses of biological examination with reference to the diagnosis of cholera. These courses are open to representatives of health boards, health officers, and to properly equipped medical men. In view of the possible advent of cholera at any time, and especially this year, this would seem to us a most valuable opportunity for health officers to obtain such technical instructions with reference to this subject as is here offered. The general scope of the courses is expected to fulfil the same purpose as the cholera courses given at the Hygienic Institute in Berlin, by Koch, in 1886 and 1887. Dr. Edward K. Dunham, who has worked considerably in cholera in Germany, and recently in this country, is conducting them. The courses began about the 20th of January, 1893, and each course continues about two weeks. The fee to cover expenses is $25. All applications for admission to the courses should be made in advance to the Secretary of the Carnegie Laboratory, 28 East Twenty-sixth street, New York.

---

THE American Text-Book of Surgery, edited by Professors Keen and White, of Philadelphia, which has only been issued a few months, is already a phenomenal success. It has been adopted as a text-book by forty-nine of our leading medical colleges and universities. Nearly 5,000 copies have been placed in physicians' libraries, and every indication points to a sale of at least as many copies more in the next six months. Dr. Nicholas Senn, of Chicago, is now preparing a Syllabus of Lectures on the Practice of Surgery, arranged in conformity with the American Text-Book of Surgery, which will be a valuable aid to all who have this great book.

---

THE Government of Venezuela and the Pan-American Medical Congress: Senor P. Ezequiel Rojas, the Venezuelean Minister of Foreign Affairs, has forwarded, on behalf of his government, through the United States *Chargé d' Affaires* at Caracas, a formal acceptance of the invitation issued pursuant to the joint resolution of the United States Congress to the various governments of the Western Hemisphere to send official delegates to the Pan American Medical Congress. The selection of delegates has not yet been made, but the names will be forwarded at the earliest possible moment.

---

THE International Medical Annual for 1893 is soon to be published by E. B. Treat, of Nos. 5 and 13 Cooper Union, New York. One of the many interesting features of this valuable work is the series of articles on special subjects connected with medicine and surgery. In looking over the Annual for 1889 we were surprised to find such exhaustive articles on Massage and Electro-Therapeutics, comprehensive enough for any practitioner or specialist. The 1893 Annual will contain several very important articles on special subjects.

This enterprising firm is now distributing to the profession in Buffalo the second edition of Illustrated Medicine and Surgery, by

Fox and Sturgis, a work of 203 pages, with half as many beautiful engravings on clinical cases by some of the foremost clinicians in America.

---

The firm of E. Leitz, of Wetzlar, Germany, manufacturers of the famous microscopes bearing this name, has established a branch office at New York, No. 30 E. 23d street.

---

St. Clement's Hospital, of Philadelphia, is to be reorganized as a hospital for epileptics, and placed under the charge of Dr. Charles K. Mills and Dr. Wharton Sinkler. It has been estimated that in the United States there are 120,000 epileptics, 1,000 to 2,000 alone in Philadelphia. We hope to see the time when not only every State but every large city will have its epileptic colony or epileptic hospital.

